# Critically appraised topic on adverse food reactions of companion animals (4): can we diagnose adverse food reactions in dogs and cats with in vivo or in vitro tests?

**DOI:** 10.1186/s12917-017-1142-0

**Published:** 2017-08-30

**Authors:** Ralf S. Mueller, Thierry Olivry

**Affiliations:** 1 Medizinische Kleintierklinik, Centre for Clinical Veterinary Medicine, LMU Munich, Veterinaerstrasse 13, 80539 Munich, Germany; 2 Department of Clinical Sciences, College of Veterinary Medicine, North Carolina State University, 1060 William Moore Drive, Raleigh, NC 27607 USA

**Keywords:** Atopic, Canine, Feline, Food allergy, Gastroenteritis, In vitro, Serum test, IgE

## Abstract

**Background:**

The gold standard to diagnose adverse food reactions (AFRs) in the dog and cat is currently an elimination diet with subsequent provocation trials. However, those trials are inconvenient and client compliance can be low. Our objective was to systematically review the literature to evaluate in vivo and in vitro tests used to diagnose AFR in small animals.

**Results:**

We searched three databases (CAB Abstracts, MEDLINE and Web of Science) for pertinent references on September 16, 2016. Among 71, 544 and 41 articles found in the CAB Abstract, MEDLINE and Web of Science databases, respectively, we selected 22 articles and abstracts from conference proceedings that reported data usable for evaluation of tests for AFR. Serum tests for food-specific IgE and IgG, intradermal testing with food antigens, lymphocyte proliferation tests, fecal food-specific IgE, patch, gastroscopic, and colonoscopic testing were evaluated.

**Conclusions:**

Testing for serum food-specific IgE and IgG showed low repeatability and, in dogs, a highly variable accuracy. In cats, the accuracy of testing for food-specific IgE was low. Lymphocyte proliferation tests were more frequently positive and more accurate in animals with AFR, but, as they are more difficult to perform, they remain currently a research tool. All other reported tests were only evaluated by individual studies with small numbers of animals. Negative patch test reactions have a very high negative predictability in dogs and could enable a choice of ingredients for the elimination diet in selected patients. Gastroscopic and colonoscopic testing as well as food-specific fecal IgE or food-specific serum IgG measurements appear less useful. Currently, the best diagnostic procedure to identify AFRs in small animals remains an elimination diet with subsequent provocation trials.

## Background

Elimination diets with subsequent provocation trials are uniformly recommended to diagnose adverse food reactions (AFRs) in dogs and cats [1–5]. However, performing home-cooked elimination diets and monitoring of clinical changes during the diet and subsequent provocation tests are work-intensive and time consuming and pet and client compliance is variable [1, 6]. For owners, laboratory tests of blood, saliva, and hair from patients offer an easier way to achieve a diagnosis of AFRs.

## Clinical scenario

Consider the example of two patients: a six-month-old female intact Labrador retriever and a five-year-old female spayed Domestic Shorthair cat. Both animals exhibit pruritus that manifests by year-round scratching. The dog also suffers from flatulence and occasional episodes of vomiting. The cat has several patches of self-induced hair loss on the abdomen and flanks and an indolent ulcer on the left upper lip. You inform the owners of both patients that you suspect that all clinical signs might be caused by a reaction to a component of their pet’s diet and advise that an elimination diet is indicated for eight weeks to evaluate potential food involvement [7]. The owners ask you if there is an easier way to identify the role of food antigens such as, for example, a blood test.

## Structured question

*Can we diagnose AFRs in dogs and cats with* in vivo *or* in vitro *tests?*

## Search strategy

We searched the Web of Science (Core Collection), MEDLINE and CAB Abstract databases on September 16, 2016 using the following string: (dog* or canine or cat* or feline) and (food* or diet*) and test* and (allerg* or hypersens* or adverse) not (human* or child* or adult*). We limited the search to journal articles published from 1980 to present; there were no language restrictions. Bibliographies from selected articles and proceedings of recent specialized veterinary dermatology and internal medicine conferences were also searched.

## Identified evidence

Our literature search identified 71, 544 and 41 articles in the CAB Abstract, MEDLINE and Web of Science (Core Collection) databases, respectively. Abstracts of relevant titles were screened and any potentially useful manuscript was downloaded and scrutinised in detail. The bibliography of these articles was examined further for additional pertinent citations. In addition, proceedings of recent veterinary dermatology or internal medicine conferences were evaluated.

Altogether, we selected 23 papers [1, 3–6, 8–25] and one abstract from conference proceedings [26] that reported results of various laboratory tests in dogs or cats where an AFR was definitely diagnosed or ruled-out. We excluded studies where the diagnosis of AFR was not confirmed or the results of the individual laboratory tests could not be attributed to a specific patient. The chosen publications were mostly case-control studies, and there were two case series [11, 12] and one each a single case report [10] and a prospective cohort study [13]. In all, there were twelve studies testing food-specific IgE in the serum of dogs [1, 5, 6, 9, 10, 12–14, 16, 18, 23, 24] and three in cats [15, 17, 21]. Four studies also evaluated canine food-specific IgG [1, 5, 16, 27]. Lymphocyte proliferation tests were assessed in four studies in dogs [11, 14, 18, 20] and one in cats [15]. In dogs, intradermal testing and gastroscopic food testing were reported in six [3, 4, 9, 10, 13, 14] and three studies [8, 19, 28] respectively. There were two studies for patch testing in dogs [5, 25], and one study each for gastroscopic food testing in cats [17], colonoscopic testing in dogs [22], determination of canine fecal IgE [8] and hair and saliva testing in dogs [26]. Some studies evaluated several different tests in dogs [1, 4, 5, 10, 11, 13, 14, 16, 18, 23] and in cats [15]. Studies were reported from 1991 [4] to 2017 [24, 25]}. All papers were in English except one, which was in German [1]. The number of animals and type of test performed in each paper are listed in Table 1.

**Table 1:** Number of tested animals and type of test performed

Reference	Dogs/Cats with AFR	Control dogs/cats ^a^	Type of test evaluated	Diet & provocation details known?
Allenspach et al. 2006 [22]	9 D	5 D	CT	Yes
Belova et al. [21]	15 C	114 C	IgE	No
Bethlehem et al. 2012 [5]	18 D	18 D	PT, IgG, IgE	Yes
Coyner & Schick 2016 [26]	1 D	6 D	HT, ST	No
Devaud et al. 2009 [20]	5 D	7 D	LT	Yes
Elwood et al. 1994 [19]	8 D		GT	
Favrot et al. 2017 [24]	14 D	32 D	IgE	No
Foster et al. 2003 [23]		91 D	IgE, IgG	No
Fujimura et al. 2011 [18]	13 D	12 D	IgE, LT	No
Guilford et al. 2001 [17]	16 C	39 C	GT, IgE	Yes
Hardy et al. 2014 [16]	8 D	43 D	IgG, IgE	No
Ishida et al. 2004 [14]	11 D		IDT, IgE, LT	Yes
Ishida et al. 2012 [15]	3 C		IDT, IgE, LT	Yes
Jackson et al. 2003 [13]	14 D		IDT, IgE	Yes
Jeffers et al. 1991 [4]	13 D		IDT, IgE	Yes
Johansen et al. 2017 [25]	24 D		PT	Yes
Kang et al. 2014 [12]		101 D	IgE	No
Kawano et al. 2013 [11]	12 D (no rechallenge)		IgE, LT	No
Kunkle & Horner 1992 [3]	9 D	61 D	IDT	No
Mueller & Tsohalis 1998 [6]	8 D	8 D	IgE	Yes
Ohmori et al. 2007 [10]	1 D		IDT, IgE	Yes
Puidgemenot et al. 2006 [9]	9 D	3 D	IDT	Yes
Vaden et al. 2000 [8]	6 D		GT, IgE	Yes
Wilhelm & Favrot 2005 [1]	5 D	32 D	IgE, IgG	No

*C* cat, *D* dog, *GT* gastroscopic food sensitivity testing, *HT* hair testing, *IDT* intradermal testing with food antigens, *IgG* serum testing for food-specific IgG, *IgE* serum testing for food-specific IgE, *PT* patch testing with food antigens, *ST* salivary testing

^a^healthy or allergic, but not adverse food reaction

## Evaluation of evidence

Calculations of the accuracy, positive and negative predictabilities of the various tests for a positive food challenge in dogs and cats with naturally occurring AFRs are reported in Tables 2 and 3, respectively.

**Table 2:** Accuracy, positive and negative predictability^a^ of various tests in privately owned dogs with naturally occurring adverse food reactions based on provocation with individual food allergens

	Accuracy	Positive predictability	Negative predictability
Intradermal testing with food antigens [4, 14]	63–76%	60–67%	62–77%
Serum testing for food-specific IgE [4, 5, 14]	58–87%	15–100%	61–86%
Serum testing for food-specific IgG [5]	77%	35%	84%
Lymphocyte proliferation tests [14]	94%	100%	93%
Patch testing with food antigens [5, 25]	81–90%	63–75%	88–99%

^a^ The accuracy was calculated by dividing the number of correct results by the number of all results verified, positive predictability by dividing correctly positive results by the total number of positive results and negative predictability by dividing correctly negative results by the total number of negative results

**Table 3:** Accuracy, positive and negative predictability of various tests in privately owned cats with naturally occurring adverse food reactions

	Accuracy	Positive predictability	Negative predictability
Serum testing for food-specific IgE [15]	20%	0%	20%
Lymphocyte proliferation tests [15]	80%	100%	50%
Intradermal tests [15]	47%	100%	27%

These parameters could not be evaluated in nine of the selected studies, mainly because of the lack of performance of provocation tests with individual food items [1, 11, 12, 16, 18, 19, 21, 23, 28]. One report only provided details of the individual positive, but not negative provocation trials—and this prevented the calculation of the tests’ accuracy [17]. Some studies evaluated the tests only in laboratory dogs [8, 9, 13, 20, 22], and it is not clear if the pathophysiology of AFRs in sensitized laboratory animals mirrors that of the naturally-occurring disease. In most studies using laboratory dogs, the tests were more accurate, presumably because the more controlled environment and food intake might have minimized the impact of other environmental factors that could be influencing the development of clinical signs. Some studies had only six or fewer of dogs [8, 10, 20] or cats [15] with AFRs included. While most reports were of animals with cutaneous AFRs, dogs [19, 22, 28] and cats [15, 17] with gastrointestinal disease were also included in some articles.

While testing for allergen-specific IgE is well established for environmental allergens in humans, dogs and cats [29], it is also offered for food allergens in many countries; this explains while most of our included studies evaluated serum food-specific IgE testing. Two studies showed a low repeatability of serum food-specific IgE and IgG testing when different aliquots of the same sample were evaluated in a blinded fashion [1, 16], the authors then concluded that these tests were unsuitable for clinical use. One study found a high concentration of food-specific serum IgE in a large number of dogs that had environmental atopic dermatitis and that had signs that did not improve after being fed an elimination diet [12]. Similar results were obtained in other studies in which dogs with AFRs were compared to apparently healthy dogs [5, 6, 14, 16, 18, 23]. When the serum test results for food-specific IgE were correlated with food provocation outcomes in dogs with AFRs [4–6, 9, 10, 14, 24], the tests’ accuracy and positive and negative predictabilities varied highly.

Intradermal testing with food antigens in laboratory dogs sensitized to specific foods usually yielded concordant positive reactions [9, 13]. When allergic patients in clinical practice were tested, however, dogs with environmental, but non- food-induced atopic dermatitis also exhibited numerous positive reactions to food antigens [3], while dogs with AFRs often had no positive results [3, 4, 14].

With lymphocyte proliferation tests [11, 14, 15, 18, 20], the accuracy was generally higher, but this test is technically more difficult to conduct and blood specimens need to be processed very quickly after sampling, two reasons why this test is generally not offered by commercial laboratories.

In the two studies assessing the usefulness of patch testing with food antigens, the accuracy and negative predictability of patch testing were satisfactory and excellent respectively (particularly for protein sources), but the positive predictability was low [5, 25]. As a result, this test cannot be used for the diagnosis of AFR but it could be useful as a tool to identify suitable ingredients for the elimination diet in selected dogs.

Gastroscopic testing had an unsatisfactory accuracy in dogs [8, 19, 28] and cats [17]; the same was evaluated for fecal food-specific IgE [8] and hair and saliva testing [26].

## Conclusion and implication for practitioners

Patch testing with food ingredients might be useful in some selected dogs to choose the ingredients for an elimination diet. Currently, all other tests cannot be recommended for the clinical diagnosis of AFRs in dogs and cats. Although serum IgE testing for food-specific IgE is offered by many laboratories in many countries as a tool for the diagnosis of AFRs, it is not reliable in dogs and cats. At this time, the best diagnostic procedure to identify AFRs in small animals remains an elimination diet with subsequent provocation trials.

## Abbreviations

AFR (s): Adverse food reaction (s)
CAT: Critically appraised topic

## References

[1] Wilhelm S, Favrot C (2005). Food hypersensitivity dermatitis in the dog: diagnostic possibilities. Schweiz Arch Tierheilkd.

[2] Proverbio D, Perego R, Spada E, Ferro E (2010). Prevalence of adverse food reactions in 130 dogs in Italy with dermatological signs: a retrospective study. J Small Anim Pract.

[3] Kunkle G, Horner S (1992). Validity of skin testing for diagnosis of food allergy in dogs. J Am Vet Med Assoc.

[4] Jeffers JG, Shanley KJ, Meyer EK (1991). Diagnostic testing of dogs for food hypersensitivity. J Am Vet Med Assoc.

[5] Bethlehem S, Bexley J, Mueller RS (2012). Patch testing and allergen-specific serum IgE and IgG antibodies in the diagnosis of canine adverse food reactions. Vet Immunol Immunopathol.

[6] Mueller RS, Tsohalis J (1998). Evaluation of serum allergen-specific IgE for the diagnosis of food adverse reactions in the dog. Vet Dermatol.

[7] Olivry T, Mueller RS, Prelaud P (2015). Critically appraised topic on adverse food reactions of companion animals (1): duration of elimination diets. BMC Vet Res.

[8] Vaden SL, Hammerberg B, Davenport DJ, Orton SM, Trogdon MM, Melgarejo LT (2000). Food hypersensitivity reactions in soft coated wheaten terriers with protein-losing enteropathy or protein-losing nephropathy or both: gastroscopic food sensitivity testing, dietary provocation, and fecal immunoglobulin E. J Vet Int Med..

[9] Puigdemont A, Brazis P, Serra M, Fondati A (2006). Immunologic responses against hydrolyzed soy protein in dogs with experimentally induced soy hypersensitivity. Am J Vet Res.

[10] Ohmori K, Masuda K, Kawarai S, Yasuda N, Sakaguchi M, Tsujimoto H (2007). Identification of bovine serum albumin as an IgE-reactive beef component in a dog with food hypersensitivity against beef. J Vet Med Sci.

[11] Kawano K, Oumi K, Ashida Y, Horiuchi Y, Mizuno T (2013). The prevalence of dogs with lymphocyte proliferative responses to food allergens in canine allergic dermatitis. Pol J Vet Sci.

[12] Kang MH, Kim HJ, Jang HJ, Park HM (2014). Sensitization rates of causative allergens for dogs with atopic dermatitis: detection of canine allergen-specific IgE. J Vet Sci.

[13] Jackson HA, Jackson MW, Coblentz L, Hammerberg B (2003). Evaluation of the clinical and allergen specific serum immunoglobulin E responses to oral challenge with cornstarch, corn, soy and a soy hydrolysate diet in dogs with spontaneous food allergy. Vet Dermatol.

[14] Ishida R, Masuda K, Kurata K, Ohno K, Tsujimoto H (2004). Lymphocyte blastogenic responses to inciting food allergens in dogs with food hypersensitivity. J Vet Int Med..

[15] Ishida R, Kurata K, Masuda K, Ohno K, Tsujimoto H (2012). Lymphocyte blastogenic responses to food antigens in cats showing clinical symptoms of food hypersensitivity. J Vet Med Sci.

[16] Hardy JI, Hendricks A, Loeffler A, Chang Y-M, Verheyen KL, Garden OA (2014). Food-specific serum IgE and IgG reactivity in dogs with and without skin disease: lack of correlation between laboratories. Vet Dermatol.

[17] Guilford WG, Jones BR, Markwell PJ, Arthur DG, Collett MG, Harte JG (2001). Food sensitivity in cats with chronic idiopathic gastrointestinal problems. J Vet Int Med.

[18] Fujimura M, Masuda K, Hayashiya M, Okayama T (2011). Flow cytometric analysis of lymphocyte proliferative responses to food allergens in dogs with food allergy. J Vet Med Sci.

[19] Elwood CM, Rutgers HC, Batt RM (1994). Gastroscopic food sensitivity testing in 17 dogs. J Sm Anim Pract.

[20] Devaud N, Hall JA, Gaschen F, Vallan C, Doherr MG, Williamson L (2009). Lymphocyte blastogenic response to ovalbumin in a model for canine allergy. Vet J.

[21] Belova S, Wilhelm S, Linek M, Beco L, Fontaine J, Bergvall K (2012). Factors affecting allergen-specific IgE serum levels in cats. Can J Vet Res.

[22] Allenspach K, Vaden SL, Harris TS, Grone A, Doherr MG, Griot-Wenk ME (2006). Evaluation of colonoscopic allergen provocation as a diagnostic tool in dogs with proven food hypersensitivity reactions. J Small Anim Pract.

[23] Foster AP, Knowles TG, Moore AH, Cousins PD, Day MJ, Hall EJ (2003). Serum IgE and IgG responses to food antigens in normal and atopic dogs, and dogs with gastrointestinal disease. Vet Immunol Immunopathol.

[24] Favrot C, Linek M, Fontaine J, Beco L, Rostaher A, Fischer N (2017). Western blot analysis of sera from dogs with suspected food allergy. Vet Dermatol.

[25] Johansen C, Mariani C, Mueller RS. Evaluation of canine adverse food reactions by patch testing with single proteins, single carbohydrates and commercial foods. Dig Vet Dermatol. 2017. doi:10.1111/vde.12455. 10.1111/vde.12455 28544017

[26] Coyner K, Schick A (2016). Inaccuracies of a hair and saliva test for allergies in dogs. Vet Dermatol.

[27] Lowrie M, Garden OA, Hadjivassiliou M, Harvey RJ, Sanders DS, Powell R (2015). The clinical and serological effect of a gluten-free diet in border terriers with Epileptoid cramping syndrome. J Vet Int Med.

[28] Olsen J, Guilford GW, Strombeck DR. Clinical use of gastroscopic food sensitivity testing in the dog. In: Annual conference of the American College of Veterinary Internal Medicine: 1991; New Orleans; 1991: 888.

[29] Mueller RS, Janda J, Jensen-Jarolim E, Rhyner C, Marti E (2016). Allergens in veterinary medicine. Allergy.

